# Piezoresistive Effect: A New Concept for Hearing Aids

**DOI:** 10.1002/advs.202501227

**Published:** 2025-04-11

**Authors:** Mengyao Gao, Weijie Liu, Kun Chen, Huili Sun, Xiaoqing Liu, Haonan Xing, Huatang Wang, Benpeng Zhu, Haizhong Guo

**Affiliations:** ^1^ Key Laboratory of Materials Physics Ministry of Education School of Physics Zhengzhou University Zhengzhou 450001 China; ^2^ School of Integrated Circuits Wuhan National Laboratory for Optoelectronics Huazhong University of Science and Technology Wuhan 430074 China; ^3^ Institute of Quantum Materials and Physics Henan Academy of Sciences Zhengzhou 450046 China

**Keywords:** hearing aid, MPSS, MXene, piezoresistive effect

## Abstract

Hearing loss is among the most prevalent sensory impairments globally. Hearing aids assist individuals with hearing impairments in perceiving sound more effectively, serving as essential tools for reconnecting them with the world. Herein, an MXene/Polyvinyl Alcohol sound sensor (MPSS) capable of recognizing weak sound signals is developed. It can not only recognize different speech signals and sound properties but also show good stability in the process of long‐term sound recording. In addition, by combining the sensor with machine learning, the accuracy rate of voiceprint recognition of wildlife conservation is up to 99%, underscoring its practical significance for wildlife protection. Utilizing piezoresistive effects, a hearing aid with a detection sound threshold of 60 dB and a frequency range of 20–4000 Hz is achieved, which provides a feasible scheme in hearing aids.

## Graphical abstract

An MXene/PVA sound sensor utilizing piezoresistive effects has been proposed with a detection limit of 60 dB and a frequency response of 20–4000 Hz, which is comparable to the performance of commercial hearing aids. By combining the sensor with machine learning, the accuracy rate of voiceprint recognition of wildlife conservation can reach up to 99%.

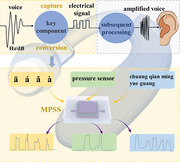

## 1 Introduction

As a vital information carrier, sound plays an essential role in human communication, environmental perception, and other aspects of daily life.^[^ ^1^, ^2^ ^]^ However, hundreds of millions of people worldwide are currently affected by hearing loss to varying degrees.^[^ ^3^, ^4^ ^]^ Population growth and aging have intensified the prevalence of hearing loss, making it an important public health issue that not only hinders communication but also adversely impacts mental health and cognitive function.^[^ ^5^, ^6^ ^]^ Hearing aids significantly improve auditory perception for patients, with their core technology centered on the capture and conversion of sound. Although the existing technology is basically matured, developing new materials, new processes, and new technologies to improve the performance of hearing aids is still very necessary.^[^ ^7^, ^8^ ^]^ Among various types of sensors, the sensor based on the piezoresistive effect has the advantages of a simple manufacturing process, low cost and power consumption, high sensitivity, and wide measurement range.^[^ ^9^, ^10^ ^]^ Furthermore, its high integration potential, good flexibility, and excellent sensing ability has attracted extensive attention in the fields of electronic skin, the Internet of Things, human‐machine interface, and so on.^[^ ^11^, ^12^, ^13^, ^14^, ^15^ ^]^ As a kind of pressure wave generated by the vibration of objects, sound can also be detected and converted into electrical signals by highly sensitive pressure sensors.^[^ ^16^, ^17^ ^]^ However, the application of sensors to hearing aids and the improvement of their minimum decibel detection limit and frequency response range has rarely been mentioned.

The special structure and properties of 2D materials make them efficient and sensitive sensing materials, among which MXene is a very competitive sensitive material in flexible pressure sensors.^[^ ^18^, ^19^, ^20^, ^21^ ^]^ MXene is a generic term of transition metal nitrides, carbides or carbon‐nitrides (the chemical formula can be expressed as M_n+l_X_n_T_x_, n = 1, 2, 3), where M represents the transition metal elements (such as Ti, Cr, Mn, and so on), X is the C or N element, and T_x_ represents the functional groups that are introduced to the surface of the MXene during etching (including ─OH, ─F, ─O, ─Cl, etc.).^[^ ^22^, ^23^, ^24^ ^]^ As a kind of typical MXene material, Ti_3_C_2_T_x_ shows high conductivity, large specific surface area, excellent mechanical properties, and adjustable interlayer spacing,^[^ ^25^, ^26^, ^27^, ^28^, ^29^, ^30^ ^]^ enabling its application in energy storage, catalysis, electromagnetic shielding, sensors, and other fields.^[^ ^31^, ^32^, ^33^, ^34^, ^35^, ^36^ ^]^ Additionally, MXene can easily form flexible self‐supporting conductive film while preserving its original properties without any surfactants. These superiorities make MXene an ideal sensitive material for the construction of highly sensitive piezoresistive pressure sensors for sound detection. Currently, some MXene‐based sound sensors based on piezoresistive effects have been applied to human sound detection and speech recognition.^[^ ^37^, ^38^, ^39^ ^]^

To enhance sensor properties such as sensitivity, low detection limit, and wide frequency response range, it is necessary to adopt strategies to optimize the sensor structure. Previous studies have proposed various microstructurally sensitive layers, such as micro‐pyramid structure, bionic microstructure, micro‐column array, micro‐hemisphere array, and so on.^[^ ^40^, ^41^, ^42^ ^]^ Through the design of the microstructure, the pressure is concentrated on the top of the microstructure, so that the contact area increases rapidly under low‐pressure conditions. Furthermore, introducing an isolation layer between the sensitive layer and the electrode further enhances the performance of the sensor.^[^ ^43^, ^44^ ^]^ It can effectively isolate the conductive paths between the sensitive layer and electrode, causing a dramatic increase in the internal conductive paths when pressure is applied.

Herein, a method of utilizing an MXene‐based pressure sensor based on piezoresistive effect to detect sound signals and serve in hearing aids is proposed. The MXene/Polyvinyl Alcohol sound sensor (MPSS) was fabricated with Polyvinyl Alcohol nanowire (PVANW) as an isolated layer sandwiched between spinosum MXene film and interdigital electrodes. Benefiting from the synergistic effect of the spinosum MXene film and the PVANW isolation layer, the sensor shows a high sensitivity of 186.65 kPa^−1^, rapid response/recover time (51.40/35.14 ms), and excellent stability under 20000 loading/unloading cycles. In sound detection, the sensor can recognize not only different sound attributes such as volume and tone, but also the structural units of different languages. Combining the MPSS with machine learning, the accuracy of voiceprint recognition for wild‐protected animals reaches 99%, demonstrating potential application prospects in wildlife protection too. Furthermore, when applied to hearing aids, a detection limit of ≈60 dB and a broad frequency response in the range of 20–4000 Hz were obtained, which can be comparable to the performance of commercial hearing aids.

## 2 Results and Discussion

### 2.1 General Concept

**Figure 1:**
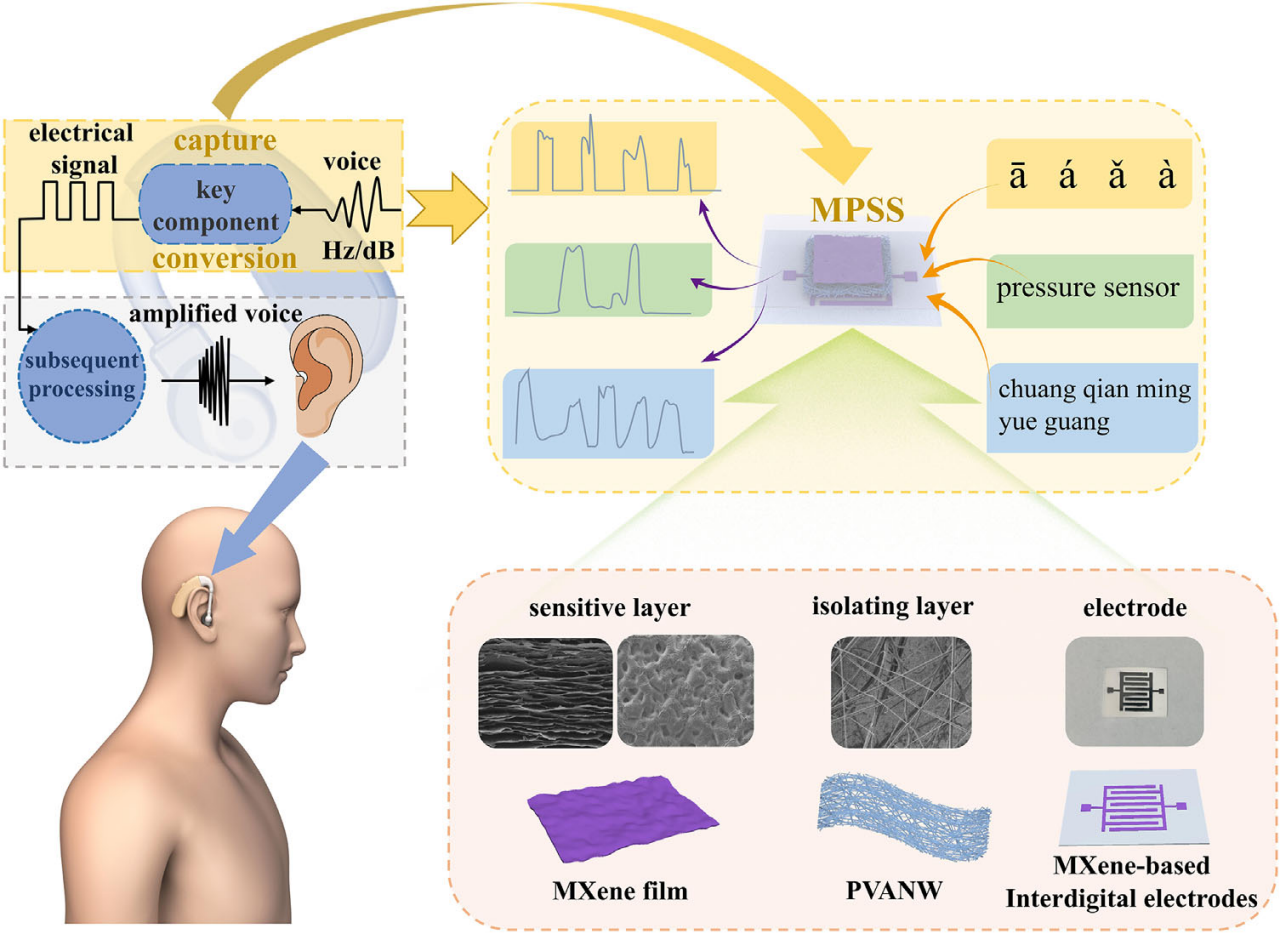
The composition of the MPSS and its potential application in hearing aids.

Hearing aids are specialized devices designed to collect and amplify sound signals for individuals with hearing impairments. The core of the device is to efficiently capture sound signals from the surrounding environment and convert these acoustic vibrations into electrical signals. Subsequently, these electrical signals are amplified through gain processing by the amplifier and re‐converted into audible sound by the receiver, which is transmitted directly to the ear. Sound, as a kind of pressure wave caused by the vibration of the object, can also be captured by a high‐sensitivity pressure sensor to realize the conversion process from sound to electrical signal, which is expected to be applied to hearing aids. In the case of the MPSS, the two‐stage amplification of the sound signal is realized through the design of the spinosum structures of MXene film and the PVANW isolation layer, enabling excellent sound sensing capability (**Figure** **1**).

The preparation of MXene nanosheets is shown in Figure S1 (Supporting Information), the mixed solution of HCl and LiF was used to selectively etch the Al atomic layer from MAX phase (Ti_3_AlC_2_) to obtain multi‐layer Ti_3_C_2_T_x_ MXene,^[^ ^45^, ^46^ ^]^ followed by ultrasonic stripping in a nitrogen atmosphere to achieve single‐layer MXene nanosheets. The prepared MXene colloidal solution appears dark green, and the Tindal effect can be observed when the laser is irradiated through it (Figure S2, Supporting Information). Transmission electron microscopy (TEM) and atomic force microscope (AFM) were used to characterize the morphology of MXene nanosheets as shown in Figure S3 (Supporting Information), the lateral size of MXene nanosheet is ≈1 µm and the thickness is ≈2.19 nm. Figure S4 (Supporting Information) presents the X‐ray diffraction (XRD) pattern and Raman spectra of MXene, further confirming the successful synthesis of Ti_3_C_2_T_x_ nanosheets.^[^ ^47^, ^48^ ^]^ The specific fabrication procedure of the MPSS is shown in Figure S5 (Supporting Information). Figure S6 (Supporting Information) shows the optical images of the spinosum MXene film, the MXene‐based interdigital electrodes, the MPSS, and their bending test, which shows good flexibility.

### 2.2 Sensing Property and Sound Detection Ability of the MPSS

Herein, six kinds of abrasive papers with different roughness are selected to fabricate MXene film with different spinosum structures (denoted as nos.100, 180, 280, 400, 600, and 800). Scanning electron microscope (SEM) images show that the spinosum structures become progressively more compact from no.100 to no.800 (Figure S7a–f, Supporting Information). And the energy‐dispersive X‐ray (EDX) mapping images show that MXene is evenly distributed on the spinosum MXene film (Figure S7g, Supporting Information). The cross‐sectional SEM images of the spinosum MXene film are shown in Figure S8 (Supporting Information), the single‐layer MXene nanosheets are stacked together with a certain interlayer spacing. Under external force, the interlayer spacing decreases, causing the nanosheets to slide or stack, which alters the conductive path and results in changes in the resistance of the sensitive layer,^[^ ^49^ ^]^ thus improving the performance of the sensor. Polyvinyl Alcohol (PVA) is a kind of water‐soluble polymer with good biocompatibility, non‐toxicity, chemical stability, and good spinnability, which is one of the most studied polymers in the field of electrospinning.^[^ ^50^ ^]^ In this work, different densities of PVANW were obtained by controlling the spinning time. Figure S9 (Supporting Information) shows the SEM images of the PVANW with spinning times of 20, 30, and 40 min, respectively. It can be seen that the densities of the PVANW increase as the spinning time increases.

**Figure 2:**
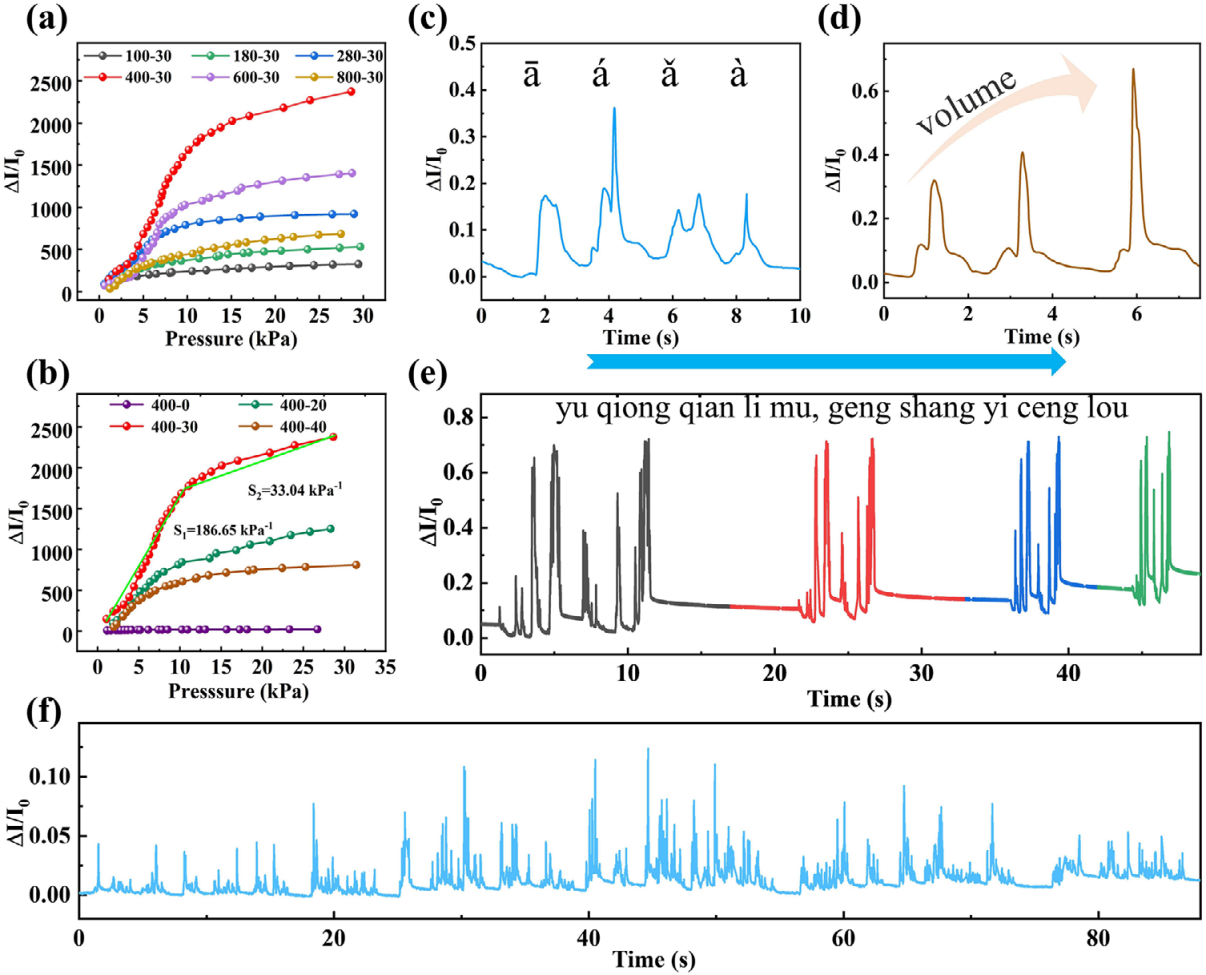
Sound sensing performance of the MPSS. Sensitivity curves of sensors with a) different spinosum structures and b) different spinning times. c) Current response to different tones of Chinese. d) Current response to sound at different volumes. e) Current response when playing ancient poems at 0.5, 1, 1.5, and 2 times. f) The sound signal waveform of the “Freedom or Death” speech (excerpt) recorded by the MPSS.

The MPSS is designed based on the piezoresistive effect, and its sensing performance is mainly evaluated by parameters such as sensitivity, response and recovery time, cycle stability, and sensing range. Sensitivity (*S*) is used to indicate the amplification degree of the sensor to the input signal, which is defined as:

(1) $$S=(\Delta I/I_{0})/\Delta P$$

where Δ*I* represents current changes before and after loading, *I*_0_ is the initial current without loading, and Δ*P* represents the change in applied pressure. Herein, the main factors affecting the sensitivity of the sensor are the different spinosum structures of the MXene film and the densities of PVANW. First, the sensitivity of sensors with different spinosum structures of the MXene films (nos.100, 180, 280, 400, 600, and 800) was tested, with spinning time controlled to be consistent across all samples. It was found that the sensor with no.400 MXene film exhibits the highest sensitivity (**Figure** **2**a). Second, Figure 2b further describes the sensitivity of the sensors with no.400 MXene film at different spinning times (0, 20, 30, and 40 min). The results show that the sensor exhibits the highest sensitivity with the no.400 MXene film and 30 min spinning time, which can be divided into two linear intervals: *S_1_* = 186.65 kPa^−1^ (<10.20 kPa), *S_2_* = 33.04 kPa^−1^ (10.20–28.60 kPa). All subsequent performance tests were conducted based on the sensor with the no.400 MXene film and 30 min spinning time. The sensor shows rapid response and recover time of 51.40 ms and 35.14 ms, respectively (Figure S10a, Supporting Information). In addition, the current of the sensor decays very slightly under 20000 cycles of loading and unloading, demonstrating excellent stability and durability (Figure S10b, Supporting Information). The *I‐V* curves from −1.00 to 1.00 V show a good linear relationship under different pressures as shown in Figure S10c (Supporting Information), indicating a good ohmic contact between the spinosum MXene film and the interdigital electrodes. The current response increases with the increase of pressure as shown in the *I‐T* curves (Figure S10d, Supporting Information), which indicates that the sensor can quickly identify different levels of pressure. Figure S11 (Supporting Information) further shows the remaining sensing performances of the MPSS. As shown in Figure S12 (Supporting Information), the cross‐sectional schematic diagram of the interaction between the sensitive layer and the interdigital electrodes in different states is established, and the equivalent circuit expression is provided to explain the sensing mechanism.

The excellent sensing performance of the MPSS gives it the ability to detect sound. Tones are important phonetic features of Chinese, and they distinguish different meanings by varying the pitch of syllables. The MPSS displays different current response waveforms for ā, á, ǎ, à (Figure 2c), meaning that it can distinguish the four different tones contained in the same syllable in Chinese. As sound volume increases, the current response of the MPSS is progressively enhanced without changing the waveform, which means that different volumes can be distinguished (Figure 2d). Figure S13 (Supporting Information) illustrates that different English letters and numbers correspond to unique and repeatable waveforms. Even in phrases and long sentences, each English word and Chinese character also corresponds to a unique waveform (Figure S14, Supporting Information), indicating that the sensor can identify the smallest structural unit of Chinese and English. When the ancient poem “yu qiong qian li mu, geng shang yi ceng lou” was played at different speeds, the waveform and peak of the sound signal recorded by the MPSS remained unchanged as shown in Figure 2e. It can be concluded that even if the frequency changes, the characteristic waveform of sound can still be recognized by the MPSS. Moreover, a 90‐s excerpt of the “Freedom or Death” speech is selected. The original audio is shown in Figure S15 (Supporting Information), and its sound signal waveform is accurately recorded by the MPSS (Figure 2f), which further illustrates the stability of the MPSS in practical application.

**Figure 3:**
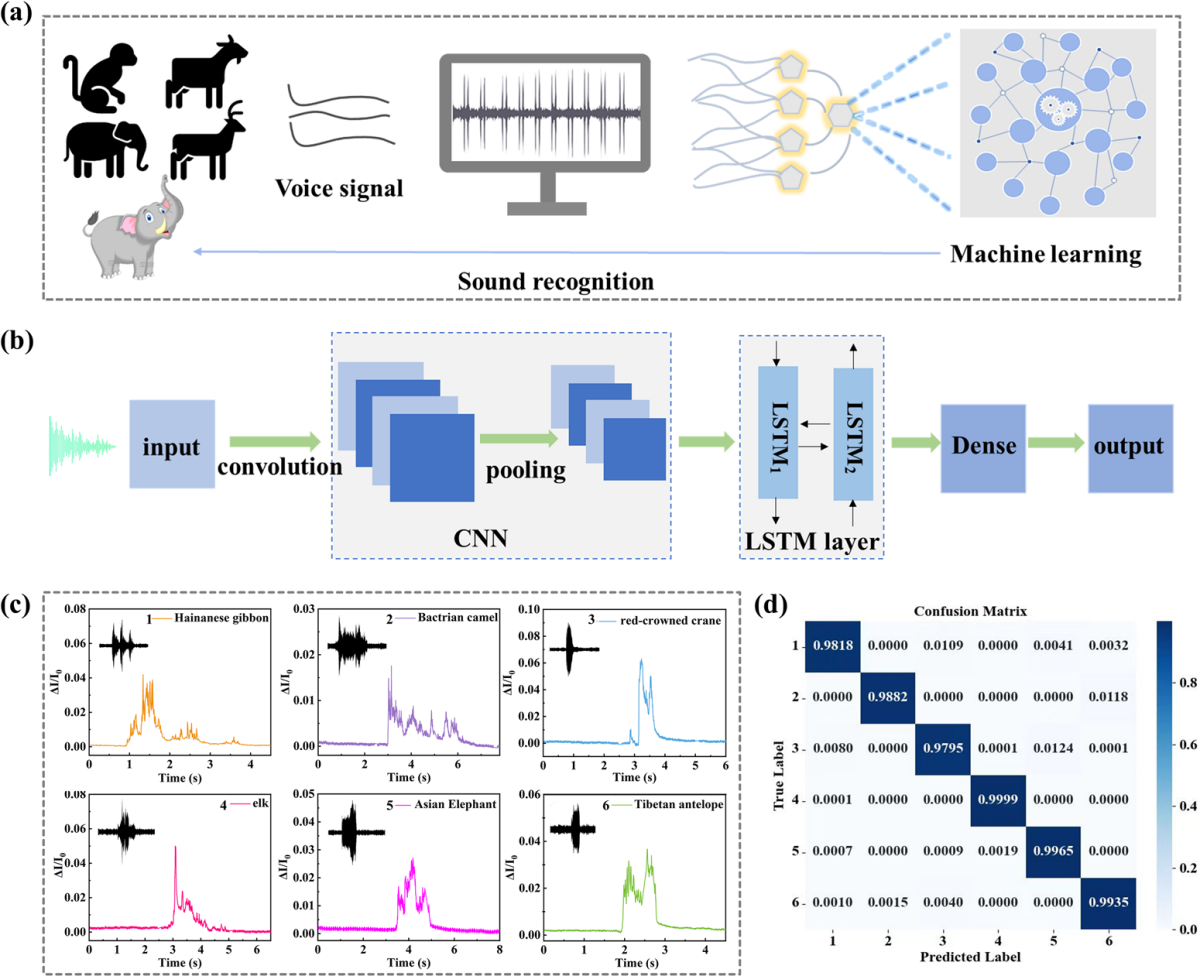
Machine learning aids animal voiceprint recognition. a) Schematic of machine learning assists the MPSS in recognizing different animal voiceprints. b) Flow chart of CNN+LSTM algorithm for animal voiceprint training and testing procedures. c) The response waveforms of six kinds of animal sounds recorded by the MPSS. The illustration is the corresponding original audio signal. d) Confusion matrix for the prediction versus the test dataset of an animal voiceprint.

Animal voiceprint recognition is an important way to detect the distribution and quantity of animals in the field, offering the advantage of not being affected by the limitation of the vision field in the wild. Deep learning simulates the working mode of the human brain by building neural networks to analyze and process data. In this work, an animal voiceprint recognition system is set up as shown in **Figure** **3**a, animal voiceprints are accurately captured by the MPSS, and a large number of data recorded by the MPSS are combined with deep learning to deeply analyze voiceprint features and obtain good recognition results. The hybrid model of convolutional neural network with long‐term and short‐term memory network (CNN+LSTM) combines the spatial feature extraction ability of CNN with the temporal modeling ability of LSTM, which has higher accuracy for complex sequence data. Figure 3b shows the flow chart of the machine learning based on the CNN+LSTM model to recognize different animal voiceprints. In the animal voiceprint recognition system, an accurate collection of animal voiceprints is essential to extract animal sound signatures.

Figure 3c records the current response waveforms of the MPSS to the sound of six endangered wildlife species, including Tibetan antelope, Asian elephant, Bactrian camel, red‐capped crane, elk, and Hainan gibbon. The unique characteristic peaks represent the voiceprints of each animal. It can be seen that the transient response waveform of the MPSS is consistent with the original audio waveform, demonstrating that it has good sound‐sensing capability. In addition, its performance is comparable to that of commercial microphone (Figure S16, Supporting Information). In the process of machine learning, 70% of the data is used as the training set, 20% as the verification set and 10% as the test set. As a result, the recognition accuracy of the obtained algorithm model can reach up to 99%. In addition, the obfuscation matrix is used to visually monitor the performance of the algorithm and measure the calibration results of the predicted and actual values. The result shows that the algorithm model achieves high recognition accuracy for each animal voiceprint (Figure 3d).

### 2.3 The MPSS in Hearing Aid

**Figure 4:**
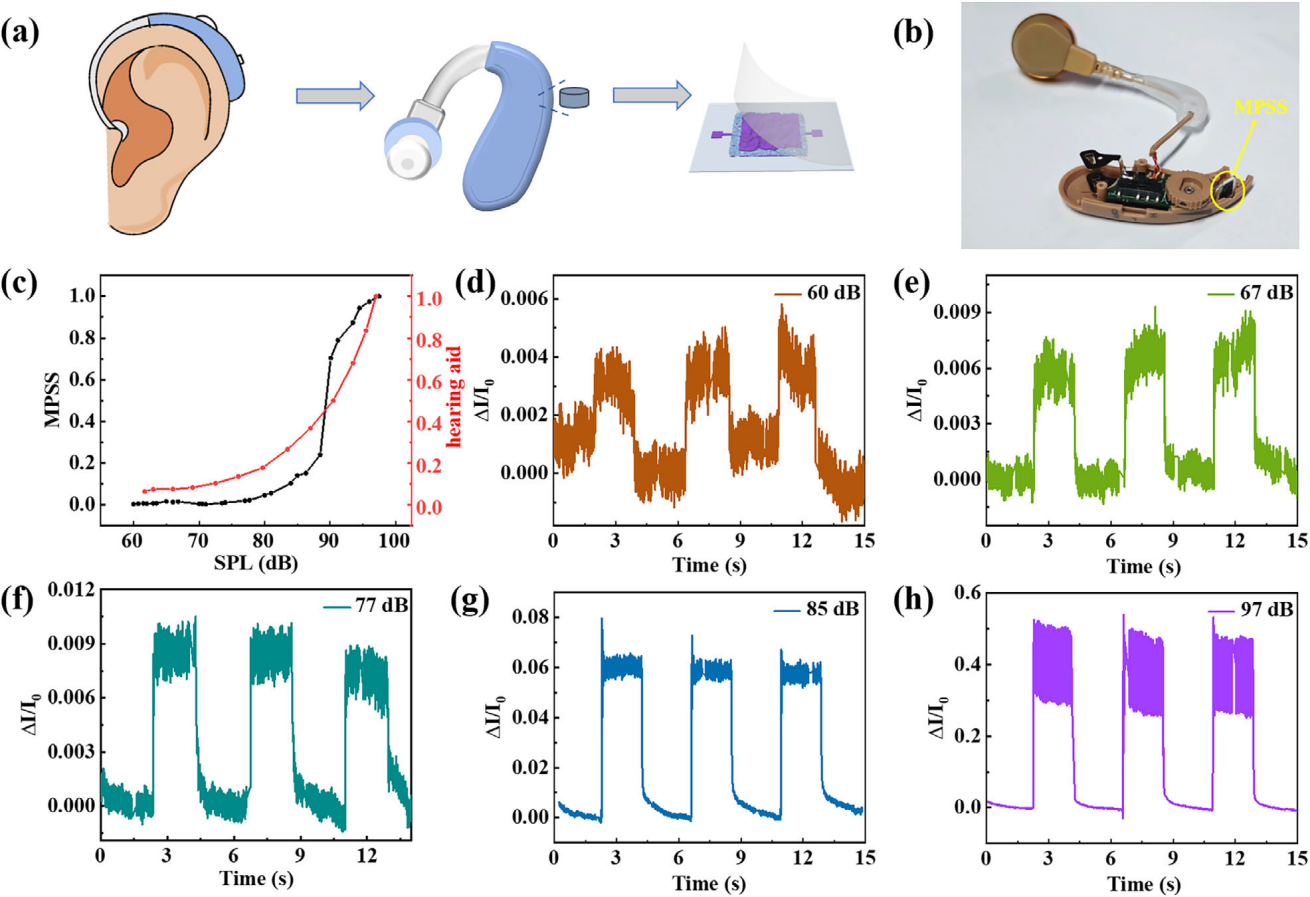
Acoustic sensing performance of the MPSS for different SPL. a) Schematic diagram of the MPSS to be applied in hearing aid. b) The optical image of the MPSS working in the hearing aid. c) Normalized current response of the MPSS and the commercial hearing aid at different SPL (the corresponding frequency is 360 Hz). d–h) The current response of the MPSS at SPL of 60, 67, 77, 85, and 97 dB.

The detection limit determines the minimum sound intensity that the hearing aid can capture. Exploring the detection limit is of great significance to optimize the hearing aid effect, adapting to different hearing loss degrees, and improving the quality of life of patients. **Figure** **4**a shows the schematic diagram of the MPSS working in the hearing aid, and the optical image is shown in Figure 4b. The sound intensity that people can feel can be quantified by the sound pressure level (SPL), which represents the logarithmic ratio of sound pressure to reference sound pressure, and can be calculated by equation:

(2) $$SPL=20Log_{10}\left(\frac{p}{p_{ref}}\right)$$

where *p* is the pressure of sound (Pa), *p_ref_* is the reference sound pressure with a value of 2×10^−5^ Pa. Figure 4c shows the variation of current response of the MPSS with SPL at a frequency of 360 Hz and compares it with the commercial hearing aid. The results show that the current response of the MPSS is positively correlated with SPL, and increases with the increase of SPL. Figure 4d–h further shows the current response curves of the MPSS in hearing aids to different SPL of 60–97 dB. It can be seen that the MPSS still shows an observable response to the sound of 60 dB.

**Figure 5:**
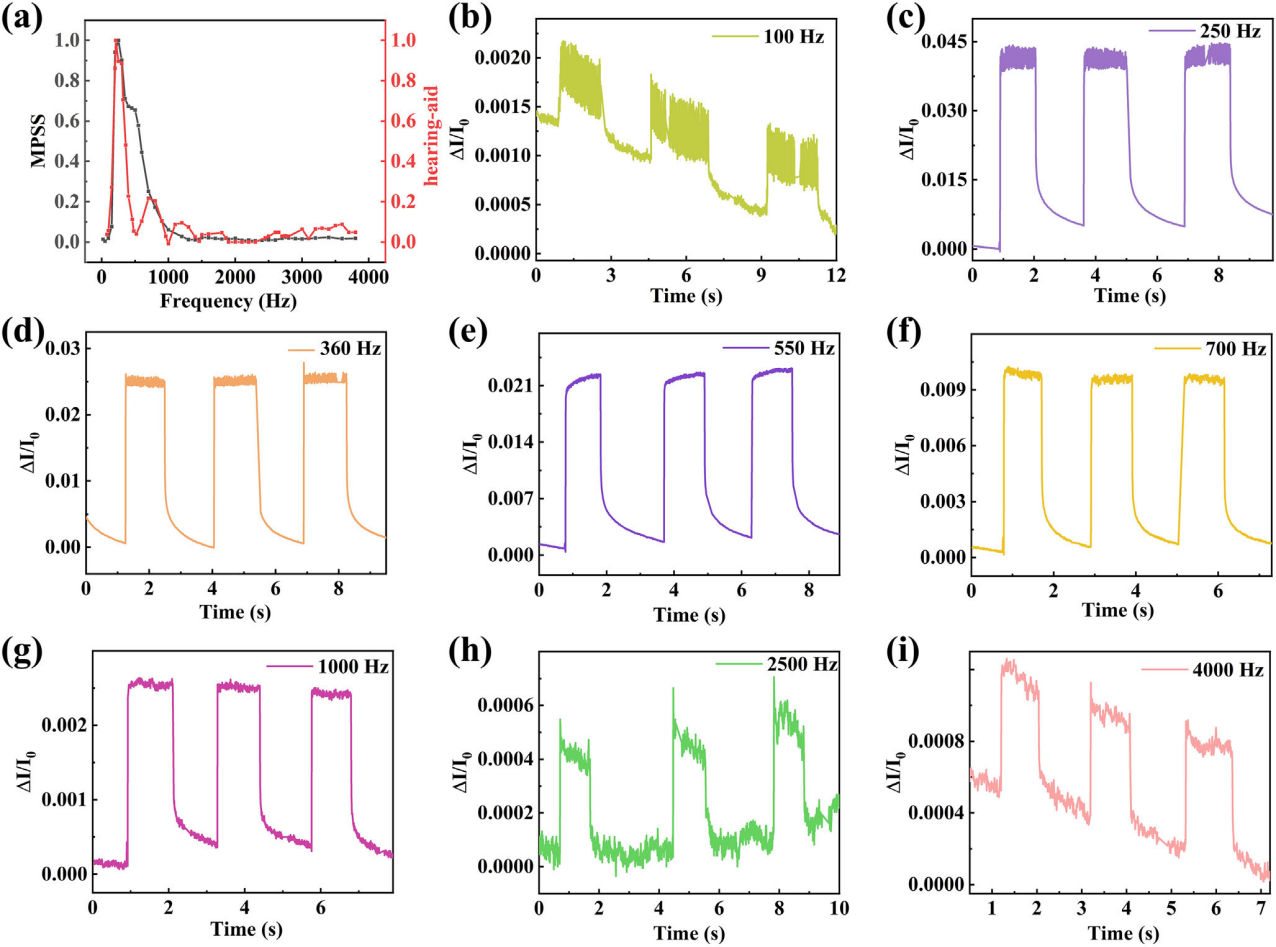
Acoustic sensing performance of the MPSS for different frequencies. a) Normalized current response of the MPSS and the commercial hearing aid at different frequencies. b–i) The current response of the MPSS at sound frequencies of 100, 250, 360, 550, 700, 1000, 2500, and 4000 Hz.

In order to further evaluate the sound sensing ability of the MPSS in the hearing aid, **Figure** **5**a shows the normalized current response of the MPSS and the commercial hearing aid at different frequencies under a voltage of 0.10 V. The output electrical response peak of the MPSS is narrow and sharp in the frequency range of 20–4000 Hz. A distinct resonant frequency is present at 250 Hz and then the response gradually decreases with the increase of frequency. The uneven frequency response of the MPSS is due to the fact that the contact resistance of the device varies slightly with the frequency. Figure 5b–i further shows the current response curves of the MPSS to different frequencies of sound. Notably, even for the low frequency of 100 Hz and the high frequency of 4000 Hz, it still shows an obvious and repeatable current response. Figure S17 (Supporting Information) shows the response of the commercial hearing aid to several different frequencies of sound. Collectively, these results show that the MPSS shows excellent sensing response to sound waves in a wide range of audible sound frequencies.

## 3 Conclusion

In this work, we prepared an MPSS with spinosum MXene film and PVANW. By designing a sensitive layer and isolation layer, the MPSS shows a high sensitivity of 186.65 kPa^−1^ and excellent sound sensing ability. It possesses the ability to identify different sound attributes. By combining a machine learning model, the recognition accuracy of animal voiceprint can reach up to 99%, which provides scientific and reasonable measures for identifying and protecting wildlife. Due to its ability to collect and convert sound through the piezoresistive effect, the MPSS has established its viability in hearing aids, and a detection sound threshold of 60 dB is obtained. It also offers a broad frequency response ranging from 20 to 4000 Hz, which covers the key frequency bands in the human daily auditory experience.

## 4 Experimental Section

### Materials

High purity Ti_3_AlC_2_ powder (400 mesh) was purchased from 11 Technology Co. LiF, and HCl were purchased from Aladdin Bio‐Chem Technology Co. and Sinopharm Chemical Reagent Co. (China), respectively. Polyvinyl alcohol was purchased from Alfa Aesar Chemical Co., Ltd. Water used in all experiments was filtered through a Laboratory water purification system (Hitech Eco‐S15, China). Chemicals were used directly without further purification.

### Preparation Process of the MXene (Ti_3_C_2_T_x_) Nanosheet Colloidal Solution

0.50 g Ti_3_AlC_2_ (MAX phase) was slowly added into the mixed solution of 0.50 g LiF and 10 mL 75% HCl, and the mixture was stirred continuously at 35 °C for 24 h. Then the reactant was centrifugally washed until pH > 6, and the supernatant of the last centrifugation should be dark green. The precipitate was dispersed in a certain amount of deionized water and sonicated for 1 h at low temperature in an inert gas atmosphere. Finally, the solution was centrifuged at 3500 rpm for 1 h, and the collected supernatant was the colloidal solution of Ti_3_C_2_T_x_ MXene nanosheets.

### Preparation of Spinosum MXene Film

A certain amount of PDMS was poured on the surface of abrasive paper, and then the PDMS substrate with spinosum structure was obtained. After that, 3 ml of MXene solution was poured on the spinosum structure of the PDMS substrate, and the MXene film with spinosum structure was obtained by peeling from the surface of the PDMS.

### Synthesis of PVA Solution and Preparation of PVANW Isolation Layer

1 g PVA powder and 50 mg sodium dodecyl benzene sulfonate (SDBS) power were added into 9 mL deionized water, and the mixture was stirred at 92 °C for 2–3 h. After that, a PVA solution with a 10% mass fraction was obtained. The PVANW was fabricated by electrospinning on interdigital electrodes with a propulsion speed of 5 µL min^−1^, an applied voltage of 20 kV, and an electrospinning time of 20–40 min.

### Manufacture of Interdigital Electrodes and Assembly of Sensor

The interdigital electrodes mask was placed on a mixed cellulose filter membrane, and the MXene colloid solution was uniformly sprayed on the filter membrane until the linear resistance per cm was less than 5.0 Ω (this process was completed on a heating table at 60 °C). The flexible MXene‐based interdigital electrodes were obtained by peeling off the mask. Two copper wires were connected to the interdigital electrodes with silver paste, and the interdigital electrodes with PVANW were placed face to face with MXene film, the MPSS was obtained by packaging them with PE at 130 °C.

### Process of Machine Learning for Animal Voiceprint Recognition

Animal voiceprint recognition includes the entire process from audio data processing, segmentation, resampling, normalization, training set, and verification set generation, to final model training and preservation. The CNN+LSTM model consists of two convolution layers, one pooled layer, two LSTM layers, and one fully connected layer. Two convolutional layers were mainly used to extract local features of data and 64 convolutional cores were used in each layer. The pooling layer was used to reduce the data dimension and retain the main feature information. Then, the model can effectively learn and capture the timing features of the sequence data through two layers of LSTM, where the second layer LSTM does not return the sequence, but outputs the state of the last time step as the input of the fully connected layer. The fully connected layer has six output nodes, corresponding to six categories of classification tasks, which were used to map the output of the LSTM to the final classification result.

### Characterization and Measurements

The morphology and thickness of MXene nanosheets were characterized by TEM (FEI Titan G2 60 300) and AFM (AIST‐NT), respectively. The structure and chemical composition of the MXene nanosheets were analyzed by a multifunctional X‐ray diffractometer (PANalytical Empyrean, Cu‐Kα) and a Raman spectrometer (LabRAMHREvo, 532.0 nm laser). The surface and cross‐section of the sensitive layer were characterized by a cold field emission SEM (JSM‐6700F). The project was reviewed by the ethics review committee of life sciences of Zhengzhou University (No. ZZUIRB2022‐35).

## Conflict of Interest

The authors declare no conflict of interest.

## Supporting information



Supporting Information

## Data Availability

The data that support the findings of this study are available from the corresponding author upon reasonable request.
