# Supplementary material for: Piezoresistive Effect: A New Concept for Hearing Aids
Source: Adv Sci (Weinh). 2025 Apr 11;12(25):2501227. doi: 10.1002/advs.202501227 (PMC12225007; doi:10.1002/advs.202501227)
Supplement: Supplementary file 1 — Supporting Information [file ADVS-12-2501227-s001.docx]

**Supporting information**

**Piezoresistive effect: a new concept for hearing aids**

*Mengyao Gao, Weijie Liu,^*^* *Kun Chen, Huili Sun, Xiaoqing Liu, Haonan Xing, Huatang Wang, Benpeng Zhu,^*^ and Hai**zhong Guo^*^*

M. Gao, W. Liu, K. Chen, H. Sun, X. Liu, H. Xing, H. Wang, H. Guo

Key Laboratory of Materials Physics, Ministry of Education, School of Physics Zhengzhou University

Zhengzhou 450001, China

E-mail: [wjliu@zzu.edu.cn](mailto:wjliu@zzu.edu.cn); [hguo@zzu.edu.cn](mailto:hguo@zzu.edu.cn)

B. Zhu

School of Integrated Circuits, Wuhan National Laboratory for Optoelectronics Huazhong University of Science and Technology

Wuhan 430074, China

E-mail: [benpengzhu@hust.edu.cn](mailto:benpengzhu@hust.edu.cn)

H. Guo

Institute of Quantum Materials and Physics, Henan Academy of Sciences

Zhengzhou 450046, China

**Note S1. Detailed description for the Figure S4.**

Figure S4a shows the X-ray diffraction (XRD) pattern of MAX phase and Ti_3_C_2_T_x_ MXene. The disappearance of (104) peak of MAX phase indicates that the Al atomic layer in Ti_3_AlC_2_ has been selectively etched successfully, and the (002) peak moves to the left indicates that the interlayer spacing increases after etching. The Raman spectra of MXene film is shown in Figure S4b, the characteristic peaks at 202 and 722 cm^-1^ correspond to A_1g_ out-of-plane vibrations of Ti and C, while the characteristic peaks at 384 and 577 cm^-1^ correspond to E_g_ and A_1g_ vibrations of O atom.

**Note S2. Detailed description for the Figure S11.**

Figure S11a and S11b illustrate the sensor can clearly distinguish pressures with different frequencies and speeds, and the output current remains stable under the same pressure. The high synchronization of *I-V* and *I-P* curves further proves that the sensor is able to respond quickly and synchronously to pressure (Figure S11c). At the same time, by weighing tiny weights of 20 mg, the sensor exhibits low detection limit (2.61 Pa) (Figure S11d).

**Note S3. Detailed description for the Figure S12.**

A simple cross-sectional schematic of the interaction between the spinosum MXene film and the interdigital electrodes is established, and an equivalent circuit model is assumed for mechanism analysis. The equivalent circuit formula is as follows:

where is the intrinsic resistances of the spinosum MXene film and the interdigital electrodes, is the contact resistance between the spinosum MXene film and the interdigital electrodes, and is the resistance produced by the extrusion of the spinosum structures of the MXene film. Initially, the PVANW is sandwiched between the spinosum MXene film and the interdigital electrodes, making only slight contact between them and a high initial resistance. Next, the contact area increase and the spinosum structure is deformed by extrusion under appropriate pressure, which leads to the change of and simultaneously. Finally, as the pressure continues to increase, the and reach saturation. This is consistent with experimental result, further proving that the synergistic effect of sensitive layer and isolation layer can improve the performance of the sensor.


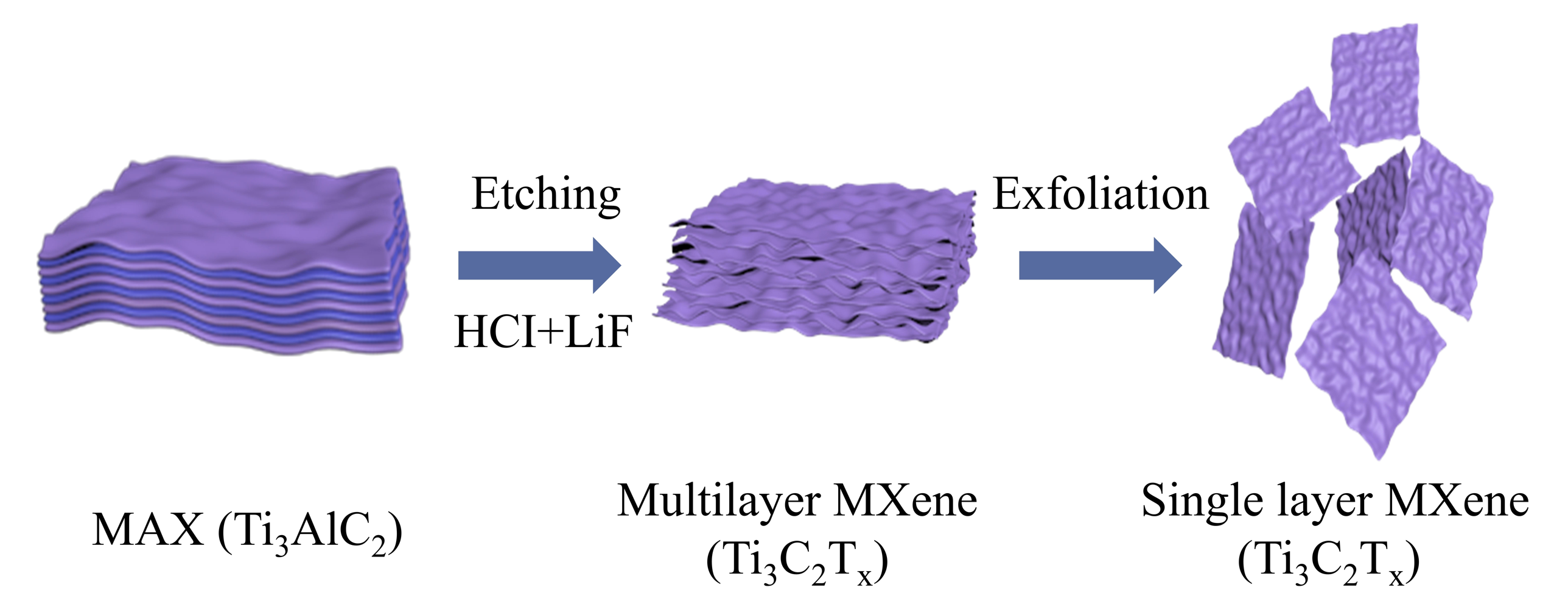


**Figure S1.** The preparation of MXene (Ti_3_C_2_T_x_) from the MAX phase (Ti_3_AlC_2_).


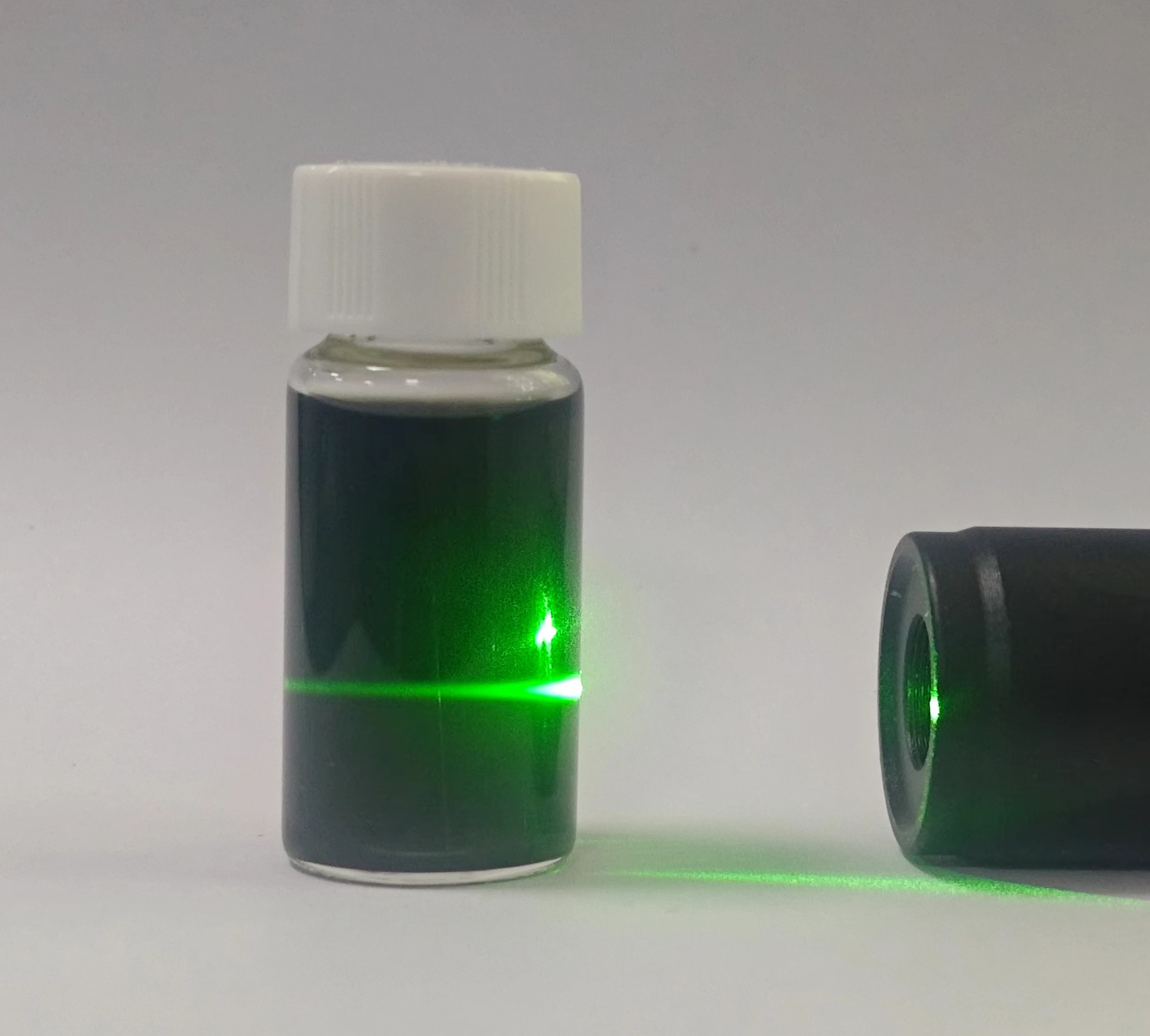


**Figure S2.** Tyndall effect of the MXene solution.


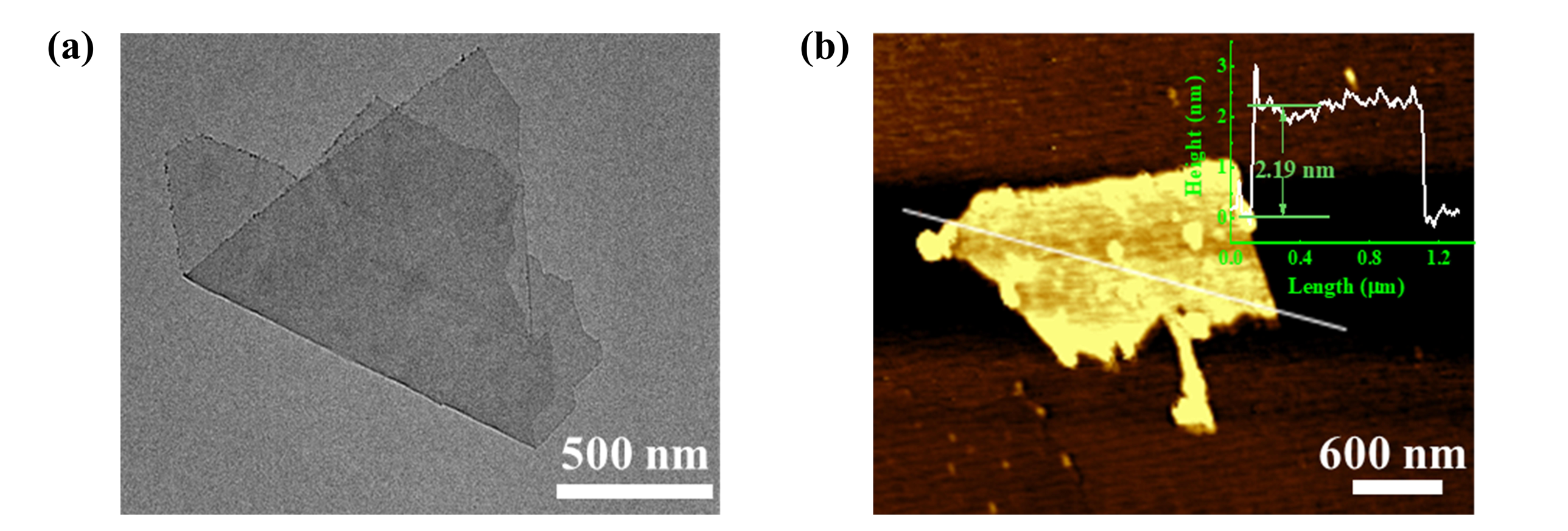


**Figure S3.** (a) TEM and (b) AFM images of the MXene nanosheet.


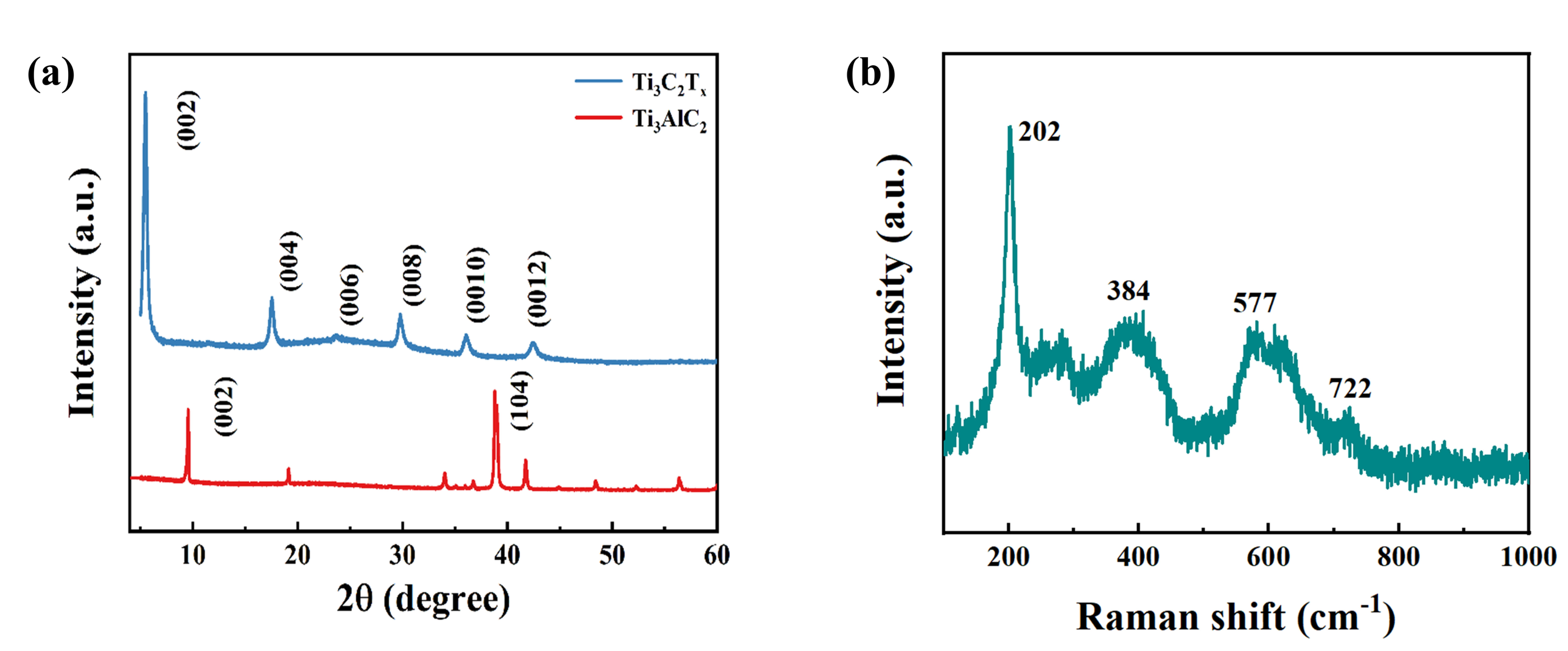


**Figure S4.** (a) XRD pattern of the MAX phase (Ti_3_AlC_2_) and Ti_3_C_2_T_x_ MXene. (b) Raman spectrum of the MXene film.


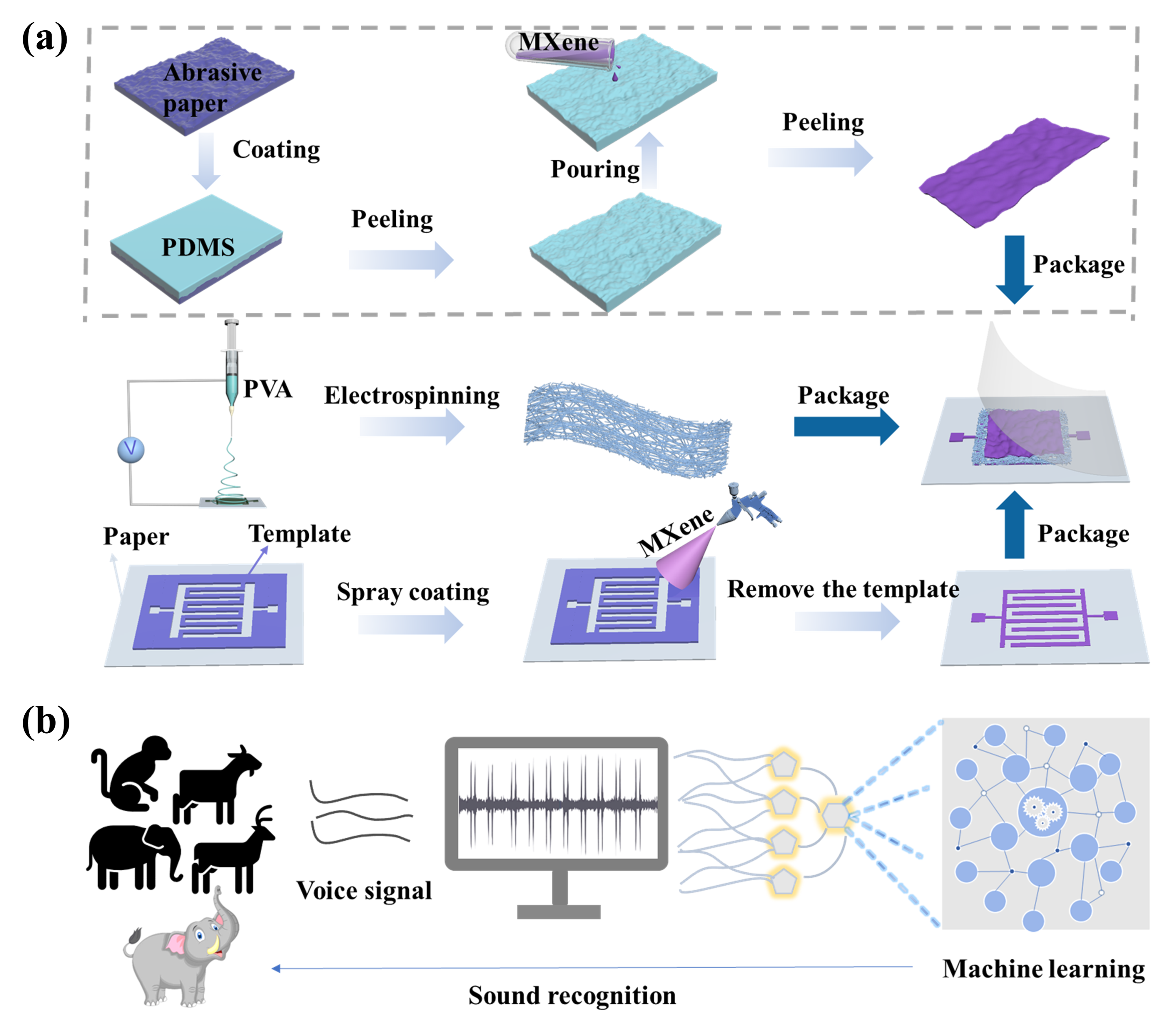


**Figure S5.** Schematic illustration of preparation of the MPSS.


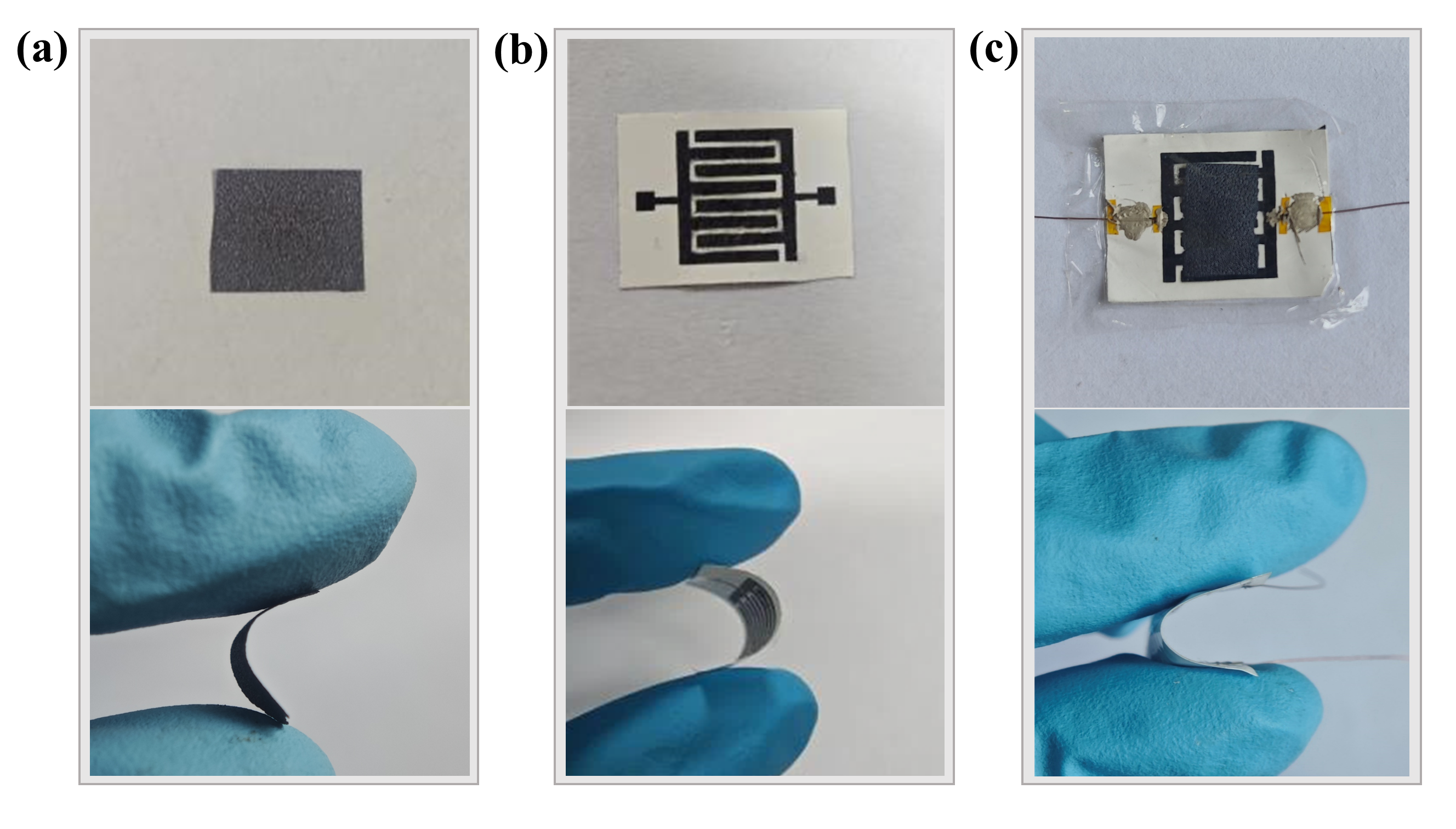


**Figure S6.** The optical images and bending tests of (a) spinosum MXene film, (b) MXene-based interdigital electrodes (mixed cellulose filter membrane was used as flexible substrate) and (c) MPSS.





**Figure S7.** (a-f) Surface SEM images of the MXene films with different spinosum structures (nos.100, 180, 280, 400, 600, and 800). (g) EDX images of the no.400 MXene film.


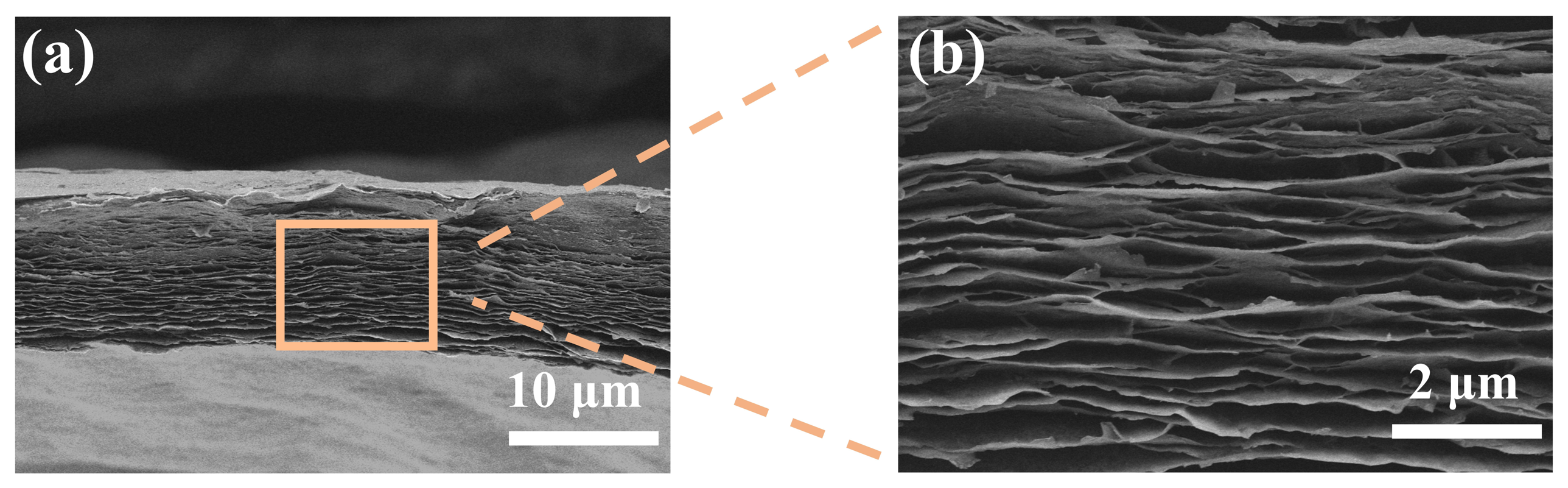


**Figure S8.** (a) Cross-sectional SEM image and (b) magnified image of spinosum MXene film.


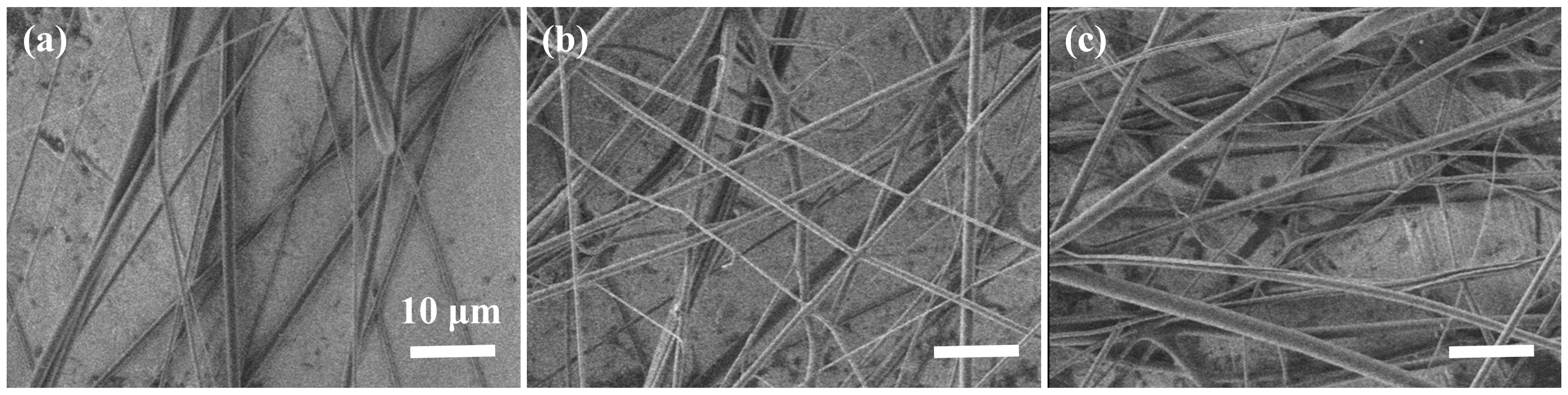


**Figure S9.** SEM images of PVANW with spinning times of (a) 20, (b) 30 and (c) 40 minutes, respectively.





**Figure S10.** (a) Response/recover time of the sensor. (b)Stability performance under 20,000 cycles of loading and unloading. (c) *I-V* curves and (d) *I-T* curves of the sensor under serial pressures.





**Figure S11.** Current response to forces with different (a) frequencies and (b) speeds. (c) The response of *I-T* and *P-T* curves under periodic loading/unloading cycles. (d) Signal response to 20, 50 and 100 mg tiny weights.





**Figure S12.** Schematic of the sensing mechanism.


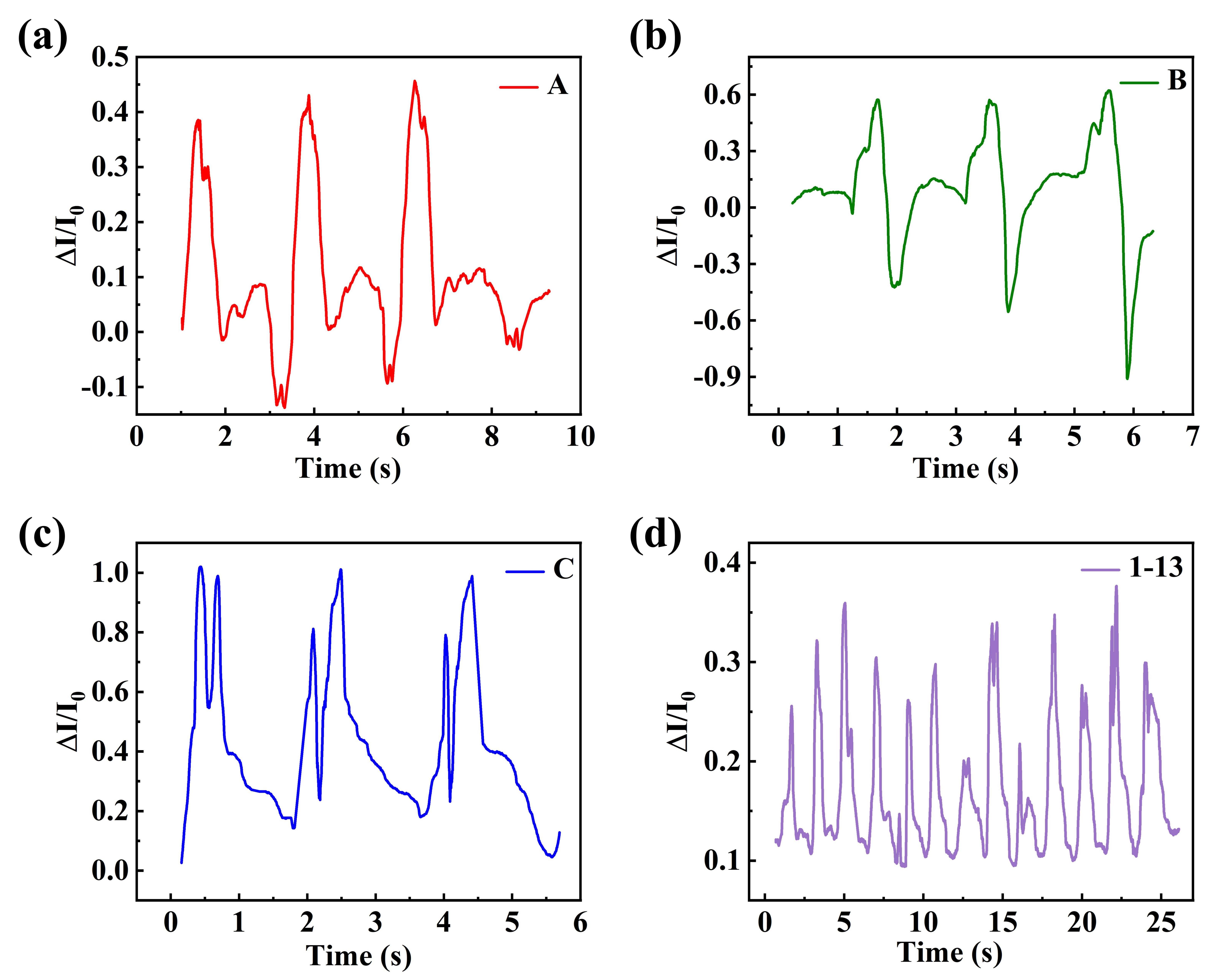


**Figure S13.** (a-d) Recognition English letters A, B, C and numbers 1-13.


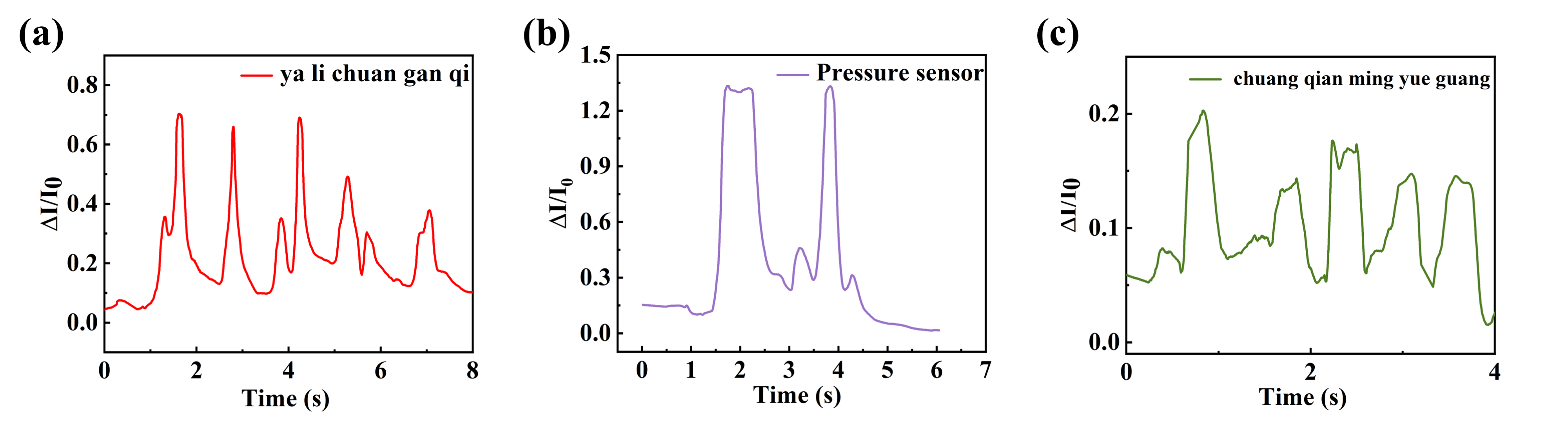


**Figure S14.** (a-c) Recognition Chinese characters and English words in phrases and sentences.


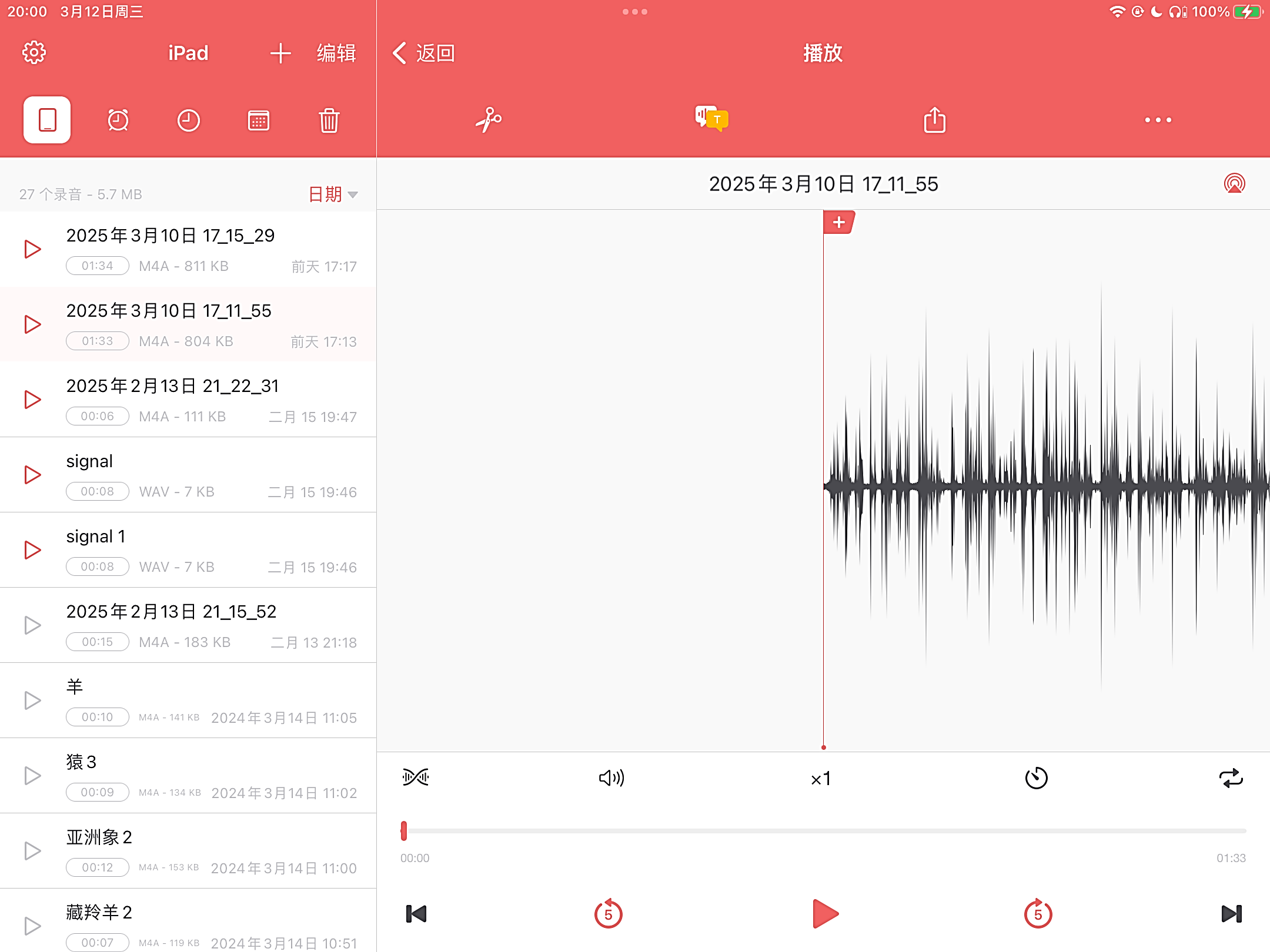


**Figure S15.** The original audio waveform of "Freedom or Death" speech (excerpt).





**Figure S16.** (a-f) The response waveforms of six kinds of animal sounds recorded by the commercial microphone.


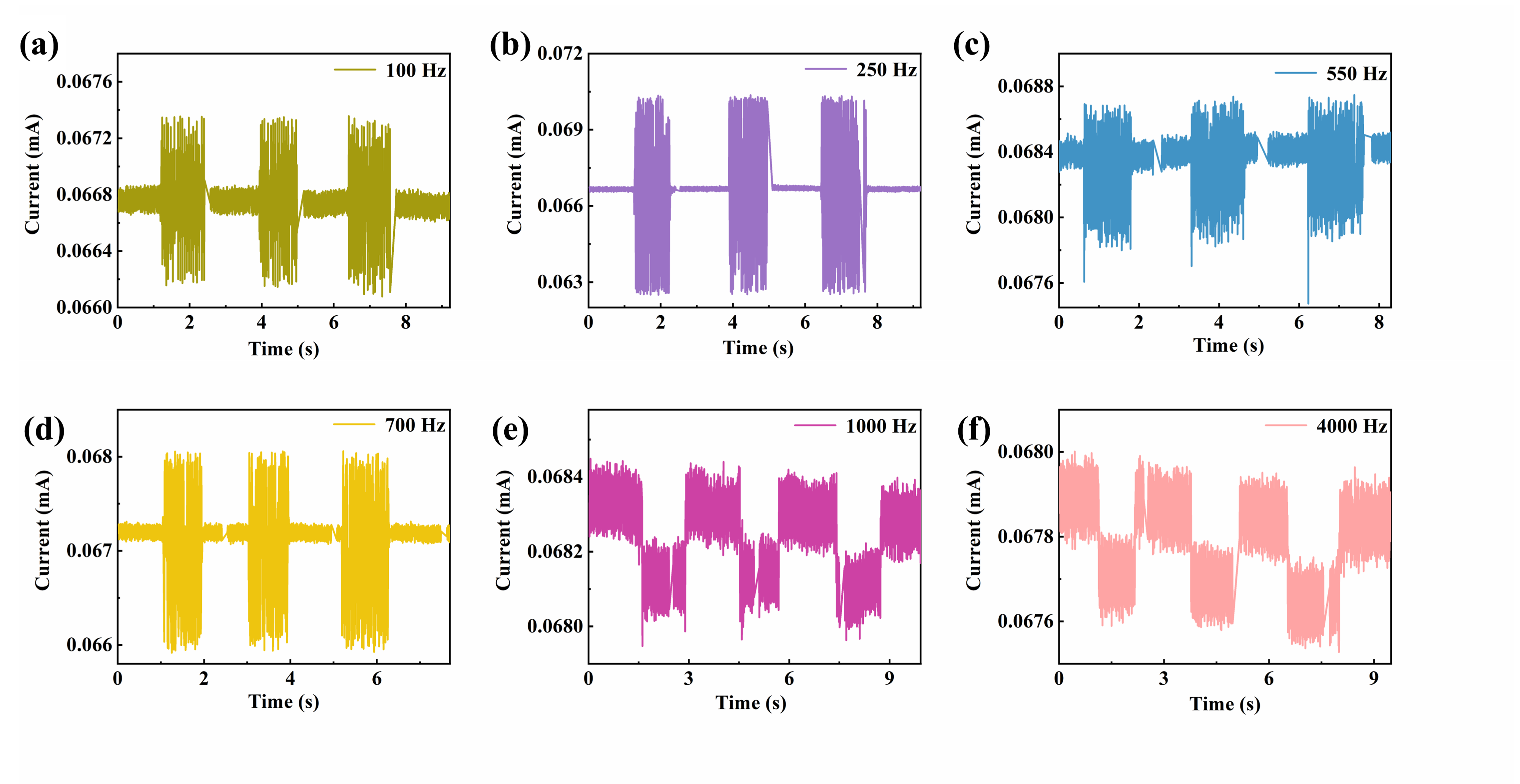


**Figure S17.** (a-f) The current response of the commercial hearing aid at frequencies of 100, 250, 550, 700, 1000 and 4000 Hz.
